# Cerebral Oximetry–Guided Anesthetic Management for Robot‐Assisted Beating‐Heart Coronary Artery Bypass Grafting in a Patient With Untreated Moyamoya Disease

**DOI:** 10.1155/cria/3262548

**Published:** 2026-05-18

**Authors:** Megumi Nagata, Miho Suzuki, Takeyuki Sajima, Atsushi Yasuda, Shigehito Sawamura

**Affiliations:** ^1^ Department of Anesthesiology, Teikyo University Hospital, 2-11-1, Kaga, Itabashi-ku, Tokyo, Japan, teikyo-u.ac.jp

**Keywords:** coronary artery spasm, moyamoya disease, off-pump, RNF213, robot-assisted coronary artery bypass grafting, rSO_2_

## Abstract

**Introduction:**

Moyamoya disease is characterized by progressive stenosis of the internal carotid arteries and impaired cerebrovascular autoregulation, posing substantial challenges during cardiac surgery. Although coronary artery bypass grafting (CABG) has been reported in patients with moyamoya disease, anesthetic management during robot‐assisted beating‐heart CABG has not been reported.

**Case Presentation:**

A 44‐year‐old woman with untreated moyamoya disease underwent robot‐assisted coronary artery bypass grafting (RACABG) for left anterior descending artery disease. One‐lung ventilation with intrathoracic carbon dioxide insufflation was required. Intraoperative anesthetic management was guided by regional cerebral oxygen saturation (rSO_2_) monitoring to ensure adequate cerebral perfusion. Strict control of blood pressure and arterial partial pressure of carbon dioxide allowed cerebral oxygenation to be maintained within 20% of baseline throughout surgery. The patient was extubated without any neurological deficits. On Postoperative Day 4, the patient developed sudden cardiac arrest, most likely due to coronary artery spasm, which lasted for approximately 19 min. She recovered fully without neurological impairment after cardiopulmonary resuscitation and percutaneous ventricular assist support.

**Conclusions:**

This case highlights the usefulness of rSO_2_‐guided anesthetic management with strict hemodynamic and carbon dioxide control during RACABG in patients with moyamoya disease. Vigilance for perioperative coronary vasospasm is essential in this population.

## 1. Introduction

Moyamoya disease is a chronic cerebrovascular disorder characterized by progressive bilateral stenosis or occlusion of the internal carotid arteries with the development of fragile collateral vessels [1, 2]. Because cerebrovascular autoregulation and carbon dioxide reactivity are often impaired [3, 4], even modest fluctuations in systemic blood pressure or arterial partial pressure of carbon dioxide (PaCO_2_) may precipitate cerebral ischemia. These features make anesthetic management challenging particularly during cardiac surgery.

Coronary artery disease has been reported in approximately 4%–5% of patients with moyamoya disease [5], and several reports have described coronary artery bypass grafting (CABG) using cardiopulmonary bypass or off‐pump techniques in this population [6–8]. Robot‐assisted coronary artery bypass grafting (RACABG) is increasingly used for selected patients with isolated left anterior descending (LAD) artery disease and offers the advantages of minimal invasiveness and reduced postoperative pain [9]. However, RACABG requires one‐lung ventilation and intrathoracic carbon dioxide insufflation, both of which may induce changes in PaCO_2_ and systemic hemodynamics that directly affect cerebral perfusion in patients with moyamoya disease [10]. To date, anesthetic management strategies for RACABG in such patients have not been reported.

We report a case of successful anesthetic management during RACABG in a patient with untreated moyamoya disease, in which regional cerebral oxygen saturation (rSO_2_) monitoring was used to guide intraoperative hemodynamic and ventilatory management, followed by an unexpected postoperative cardiac arrest with complete neurological recovery.

## 2. Case Presentation

A 44‐year‐old woman (height: 157 cm; weight: 48 kg) was diagnosed with moyamoya disease at 19 years of age. Although cerebral revascularization surgery was recommended, the patient remained under observation. During childhood, she experienced episodes of dizziness and transient loss of consciousness triggered by singing or crying. Preoperatively, the patient experienced intermittent dysarthria, numbness, dizziness, and headaches. Brain magnetic resonance imaging revealed severe bilateral internal carotid artery stenosis with abnormal collateral vessel formation and suspected old infarcts in both frontal lobes. Carotid ultrasonography showed markedly reduced internal carotid artery diameters (right: 2.2 mm; left: 2.0 mm).

One year before the current surgery, the patient developed scapular pain. Coronary angiography revealed diffuse disease in the LAD and left circumflex (LCX) arteries and complete occlusion of the proximal right coronary artery (RCA). Percutaneous coronary intervention was performed for the occluded RCA lesion. Follow‐up angiography revealed 75%–90% stenosis of the LAD artery and diffuse stenosis of approximately 50% in the RCA and LCX arteries. Transthoracic echocardiography showed a left ventricular ejection fraction of 61% with hypokinesis of the basal inferior and inferoseptal walls. Because the RCA and LCX lesions were diffuse and not suitable for revascularization and because RACABG is primarily indicated for isolated proximal LAD disease, the procedure was scheduled at the request of the surgical team.

### 2.1. Anesthetic Management

General anesthesia was induced with intravenous fentanyl (350 μg), midazolam (5 mg), and rocuronium (50 mg). After tracheal intubation with a 35‐Fr left‐sided double‐lumen tube, central venous and pulmonary artery catheters were placed via the right internal jugular vein. Standard monitoring included electrocardiography, invasive and noninvasive arterial pressure, pulse oximetry, end‐tidal carbon dioxide (PetCO_2_), central venous pressure, pulmonary artery pressure, cardiac output, cardiac index, SvO_2_, temperature, and transesophageal echocardiography. Neurological monitoring consisted of bispectral index monitoring and bilateral rSO_2_ monitoring (INVOS, Medtronic Inc., Tokyo). The primary goals of anesthesia management were to maintain stable hemodynamics and normocapnia to ensure adequate cerebral perfusion.

Baseline rSO_2_ values before induction were 77% on the right and 74% on the left. Anesthesia was maintained with sevoflurane (0.5%–1.0%) and remifentanil (0.1–0.6 μg/kg/min), with intermittent fentanyl to provide sustained analgesia and rocuronium as required. Peripheral oxygen saturation was maintained between 93% and 100%; PaCO_2_ was strictly controlled between 35 and 45 mmHg with frequent arterial blood gas analysis; and PetCO_2_ was maintained between 33 and 39 mmHg. Mean arterial pressure (MAP) and systolic blood pressure were maintained above 60 and 100 mmHg, respectively, using continuous norepinephrine infusion (0.02–0.06 μg/kg/min) with intermittent phenylephrine boluses. Hemoglobin levels were maintained above 10 g/dL, and the cardiac index was generally around 2.0 L/min/m^2^, although it transiently decreased to approximately 1.5 L/min/m^2^. Intravenous nicorandil (0.06 mg/kg/h) was administered throughout surgery.

**FIGURE 1:**
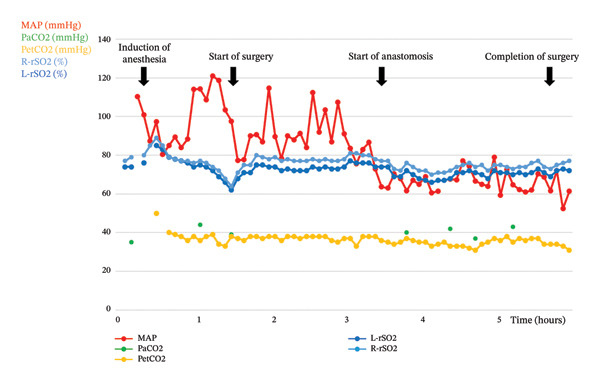
Intraoperative temporal profiles of hemodynamic and respiratory variables with concurrent cerebral oxygenation monitoring. Mean arterial pressure (MAP), arterial carbon dioxide tension (PaCO_2_), end‐tidal carbon dioxide (PetCO_2_), and bilateral regional cerebral oxygen saturation (rSO_2_; right [r] and left [l]) were recorded throughout the procedure. Immediately before surgery, initiation of remifentanil (0.2 μg/kg/min) was associated with a decrease in MAP (from 121 to 77 mmHg) and a concomitant reduction in rSO_2_ (right: 64%; left: 62%). MAP subsequently increased, accompanied by a corresponding improvement in rSO_2_. During coronary anastomosis, transient hypotension (88/39 mmHg) occurred and was associated with a decrease in rSO_2_. Phenylephrine boluses (0.05–0.1 mg) and continuous norepinephrine infusion (0.02–0.06 μg/kg/min) were administered to maintain MAP above 60 mmHg, resulting in restoration of rSO_2_ toward near‐baseline values (right: 76%; left: 71%). Throughout the procedure, rSO_2_ remained within 20% of baseline (≥ 66%).

The patient was placed in the right lateral decubitus position, and one‐lung ventilation of the right lung was instituted with carbon dioxide insufflation into the left thoracic cavity. The left internal thoracic artery was harvested with robotic assistance, followed by anastomosis to the LAD artery through a small left thoracotomy along the midclavicular line. Immediately before surgery, after initiation of remifentanil at 0.2 μg/kg/min, rSO_2_ decreased to 64% on the right and 62% on the left, in part due to a reduction in MAP from 121 mmHg to 77 mmHg (Figure 1). MAP returned to baseline subsequently without intervention, accompanied by improvement in rSO_2_. During coronary anastomosis, a transient decrease in arterial blood pressure (88/39 mmHg) was associated with a decline in rSO_2_ (right: 72%; left: 69%). Several bolus doses of phenylephrine (0.05–0.1 mg) and a continuous norepinephrine infusion (0.02–0.06 μg/kg/min) were administered to maintain MAP above 60 mmHg, resulting in restoration of rSO_2_ to near‐baseline values (right: 76%; left: 71%). Throughout the procedure, rSO_2_ remained within 20% of baseline (≥ 66%) (Figure 1). No significant electroencephalographic changes were observed, and the surgery was completed uneventfully.

### 2.2. Postoperative Course

The patient was transferred to the intensive care unit (ICU) and extubated 4 h after ICU admission without neurological deficits. rSO2 monitoring was not continued in the ICU; instead, postoperative management focused on maintaining stable hemodynamics using a pulmonary artery catheter, along with normocapnia and adequate oxygenation. On Postoperative Day 3, intravenous nicorandil was converted to oral administration upon transfer to the general ward. On Postoperative Day 4, the patient developed sudden cardiac arrest preceded by sinus pause on electrocardiography. Cardiopulmonary resuscitation was initiated immediately, and return of spontaneous circulation was achieved approximately 19 min after arrest. Coronary angiography revealed severe stenosis distal to the anastomosis, consistent with coronary vasospasm rather than thrombotic occlusion at the anastomotic site. Intracoronary nicorandil was ineffective, and balloon angioplasty was performed. Persistent cardiogenic shock necessitated insertion of a percutaneous ventricular assist device (Impella CP), and intravenous nicorandil was restarted. Neurological findings improved gradually but completely, and the patient was discharged on Postoperative Day 67.

## 3. Discussion

Moyamoya disease is a rare chronic cerebrovascular disorder with a progressive course. Over the past 3 decades, research has highlighted the importance of pain control and monitoring techniques in the perioperative management of patients with moyamoya disease because of their vulnerability to cerebral ischemia [2]. Pain induces neuroendocrine stress responses that increase cerebral metabolic demand, which is undesirable in patients with moyamoya disease due to reduced cerebral blood flow reserve [2]. Median sternotomy and cardiopulmonary bypass are more invasive and may adversely affect hemodynamics both intraoperatively and postoperatively due to postoperative pain, potentially increasing the risk of cerebral ischemia [8, 9]. Given that our cardiovascular surgeons are highly experienced in RACABG, the procedure was thus performed at their request.

This case illustrates several important anesthetic considerations for patients with moyamoya disease undergoing RACABG. First, RACABG poses unique risks because of the need for one‐lung ventilation and intrathoracic carbon dioxide insufflation, both of which can provoke fluctuations in arterial carbon dioxide tension and arterial blood pressure [10]. In patients with moyamoya disease, such changes may directly compromise cerebral perfusion because cerebrovascular autoregulation is impaired [3, 4]. In this case, rSO_2_ monitoring was used not only as an observational tool but also a real‐time guide for intervention. Hemoglobin levels and cardiac function were also closely monitored and optimized, given their influence on cerebral oxygenation. Decreases in rSO_2_ coincided with hypotension and were promptly corrected by vasopressor administration, allowing cerebral oxygenation to be maintained within 20% of the baseline throughout surgery. Strict control of PaCO_2_ was equally important because hypocapnia increases the risk for cerebral ischemia due to cerebral vasoconstriction and decreased cerebral blood flow [3], whereas hypercapnia may paradoxically reduce cerebral blood flow because of impaired cerebrovascular reactivity [3, 4]. In this patient, PaCO_2_ was carefully maintained between 35 and 45 mmHg throughout the surgery, including during periods of one‐lung ventilation, to preserve cerebral blood flow.

The postoperative cardiac arrest observed in this patient was most likely caused by a coronary artery spasm. Considering the distal location of the stenotic lesion, the clinical course leading to cardiac arrest, and the transition from intravenous to oral nicorandil, coronary artery spasm was considered the most likely cause. Patients with moyamoya disease frequently exhibit nonatherosclerotic vascular pathology characterized by intimal hyperplasia and minimal lipid deposition [11], and variants in the RNF213 gene have been associated with both moyamoya disease and coronary vasospasm [12, 13]. The transition from intravenous to oral nicorandil may have contributed to reduced vasodilatory protection, highlighting the need for continued vigilance for coronary spasm even after apparently uneventful surgery in this patient. The patient experienced a cardiac arrest lasting 19 min. Despite a prolonged cardiac arrest, the patient recovered completely without any neurological deficits. A similar case involved a 47‐year‐old woman with moyamoya disease who underwent successful off‐pump CABG but later developed cardiac arrest in the postoperative period due to coronary spasm, requiring venoarterial extracorporeal membrane oxygenation (ECMO) [14]. Both patients recovered surprisingly without any neurological deficits. Chronic cerebral hypoperfusion with collateral vessel development in moyamoya disease may confer a degree of ischemic tolerance, potentially contributing to neurological resilience in this setting [15, 16].

## 4. Conclusions

rSO_2_‐guided anesthetic management with strict control of systemic blood pressure and PaCO_2_ may facilitate safe RACABG in patients with untreated moyamoya disease. Anesthesiologists should also remain vigilant for perioperative coronary vasospasm, which may result in life‐threatening complications even after successful surgery.

### 4.1. Clinical Takeaways

Robot‐assisted beating‐heart CABG presents unique anesthetic challenges in patients with moyamoya disease because of impaired cerebrovascular autoregulation.

rSO_2_ monitoring can serve as a practical real‐time guide for maintaining adequate cerebral perfusion during one‐lung ventilation.

Strict control of PaCO_2_ and systemic blood pressure is essential to prevent cerebral ischemia.

Patients with moyamoya disease may be at an increased risk of perioperative coronary vasospasm, including those in the postoperative period.

**Nomenclature**
CABG: Coronary artery bypass grafting
RACABG: Robot‐assisted coronary artery bypass grafting
rSO_2_
: Regional cerebral oxygen saturation
PaCO_2_
: Arterial partial pressure of carbon dioxide
LAD: Left anterior descending artery
LCX: Left circumflex artery
RCA: Right coronary artery
PetCO2: End‐expiratory carbon dioxide
MAP: Mean arterial pressure
ICU: Intensive care unit
ECMO: Venoarterial extracorporeal membrane oxygenation

## Funding

No funding was received for this manuscript.

## Consent

Written informed consent for publication of this case and associated images was obtained from the patient.

## Conflicts of Interest

The authors declare no conflicts of interest.

## Data Availability

All data generated during this study are included in this published article.
